# Studying Gambling Behaviors and Responsible Gambling Tools in a Simulated Online Casino Integrated With Amazon Mechanical Turk: Development and Initial Validation of Survey Data and Platform Mechanics of the Frescati Online Research Casino

**DOI:** 10.3389/fpsyt.2020.571954

**Published:** 2021-02-05

**Authors:** Philip Lindner, Jonas Ramnerö, Ekaterina Ivanova, Per Carlbring

**Affiliations:** ^1^Department of Psychology, Stockholm University, Stockholm, Sweden; ^2^Department of Clinical Neuroscience, Centre for Psychiatry Research, Karolinska Institutet & Stockholm Health Care Services, Stockholm County Council, Stockholm, Sweden

**Keywords:** online gambling behavior, software, Amazon mechanical turk, casino gambling, Pavlovian (classical) conditioning, instrumental (operant) behavior

## Abstract

**Introduction:** Online gambling, popular among both problem and recreational gamblers, simultaneously entails both heightened addiction risks as well as unique opportunities for prevention and intervention. There is a need to bridge the growing literature on learning and extinction mechanisms of gambling behavior, with account tracking studies using real-life gambling data. In this study, we describe the development and validation of the Frescati Online Research Casino (FORC): a simulated online casino where games, visual themes, outcome sizes, probabilities, and other variables of interest can be experimentally manipulated to conduct behavioral analytic studies and evaluate the efficacy of responsible gambling tools.

**Methods:** FORC features an initial survey for self-reporting of gambling and gambling problems, along with several games resembling regular real-life casino games, designed to allow Pavlovian and instrumental learning. FORC was developed with maximum flexibility in mind, allowing detailed experiment specification by setting parameters using an online interface, including the display of messages. To allow convenient and rapid data collection from diverse samples, FORC is independently hosted yet integrated with the popular crowdsourcing platform Amazon Mechanical Turk through a reimbursement key mechanism. To validate the survey data quality and game mechanics of FORC, *n* = 101 participants were recruited, who answered an questionnaire on gambling habits and problems, then played both slot machine and card-draw type games. Questionnaire and trial-by-trial behavioral data were analyzed using standard psychometric tests, and outcome distribution modeling.

**Results:** The expected associations among variables in the introductory questionnaire were found along with good psychometric properties, suggestive of good quality data. Only 6% of participants provided seemingly poor behavioral data. Game mechanics worked as intended: gambling outcomes showed the expected pattern of random sampling with replacement and were normally distributed around the set percentages, while balances developed according to the set return to player rate.

**Conclusions:** FORC appears to be a valid paradigm for simulating online gambling and for collecting survey and behavioral data, offering a valuable compromise between stringent experimental paradigms with lower external validity, and real-world gambling account tracking data with lower internal validity.

## Introduction

Gambling refers to any activity involving wagering of money (or something of value), on an outcome that is fully or partially dependent on chance, with the possibility of winning money (or something of value). As evident by its long historical roots and popularity around the world, gambling is a popular recreational activity, often without any serious negative consequences (1). A subset of gamblers, however, develop problematic gambling behaviors such as loss-chasing, stake habituation, difficulty stopping, and gambling to escape negative emotions, and experience negative economic, psychosocial, and mental health consequences because of this (2). Gambling is now recognized as an addictive behavior in psychiatric diagnostics (3), yet unlike alcohol and substance addictions, problem gambling does not involve consuming psychoactive chemical agents. From a clinical perspective, this makes it even more important to study the specific learning and extinction mechanisms involved in gambling in order to inform gambling-specific treatment strategies, both for clinical settings and to inform so called Responsible Gambling Tools (RGT) (4).

Since the dawn of behavioral analysis, gambling has been considered a prototypical case of the effectiveness of intermittent reinforcement, wherein a behavior is rewarded some, but not all the time (5). Later behavioral analytic research has examined a broader set of learning and extinction phenomena of presumed importance to gambling (6), including other types of reinforcement schedules (7), reward discounting (8), the near-miss phenomenon (9), establishing operations (10), and verbal rules (11). Behavioral analytic research has challenged some popular preconceptions about what promotes problem gambling, e.g., revealing mixed or even contradictory evidence for the “Early Big Win” hypothesis (12–14). Recently, attempts have been made to translate these findings into clinical practice (15).

However, overall, there are surpassingly few published behavioral analytic studies of gambling behaviors given the population prevalence of both gambling and gambling problems, and its overt similarities with learning experiments (16). While the relatively small and student samples typically used in past research need not present an issue if the expected effects are large and presumed common to all humans, there is still arguably a translational need to bridge these findings with that of account tracking studies from real-life gambling, where legal requirements make it impossible to e.g., randomize participants to definitively demonstrate causality (17). Access to larger samples may also create opportunities to study even minor effects that would nonetheless have a significant public health impact. Additionally, there are surprisingly few experimental studies on specific RGT features and responsible gambling practices, given the clear policy implications and ubiquitous implementation (18).

Further, experimental studies that attempt to simulate live casino environments and games played therein, are likely to not fully capture the contextual factors that play a role in learning and extinction (19). With the advent and increasing popularity of online gambling, which is now the most prevalent type of gambling among both problem and recreational gamblers in many countries (1, 20), it is now possible to develop research paradigms that are unaffected by contextual confounders, while still accurately simulating real-life gambling. Studying learning and extinction of problem gambling behaviors in a naturalistic setting is arguably of even greater importance if the goal is to study new potential features of RGTs and responsible gambling policies in online gambling environments (21).

In the current study, we describe the development and an initial validation of the Frescati Online Research Casino (FORC): a simulated online casino where games, visual themes, outcome sizes, probabilities, and other variables can be experimentally manipulated to conduct a variety of behavioral analytic and experimental RGT research with great flexibility and convenience. Such an experimental platform would be valuable in bridging classic behavioral research and account-tracking studies on real-life gambling data, offering an attractive, translational compromise in terms of internal and external validity. Validation data was collected using an experimental setup that would allow detailed examination of the game mechanics; validity of questionnaire data was also examined using traditional psychometric techniques.

## Materials and Methods

### Amazon Mechanical Turk

Amazon Mechanical Turk (AMT) is a crowdsourcing platform that allows so called *Requesters* to publish *Human Intelligence Task* for *Workers* to complete for a pre-set monetary reimbursement. AMT has been a popular platform for collecting scientific data and running psychological experiments for many years (22–24) and has been shown to provide data of equivalent quality to traditional data collection methods (25, 26), including valid and reliable gambling data specifically (27, 28). Connecting FORC to AMT, or in principle any other crowdsourcing platform with similar features, provides access to a large, global, diverse participant pool and is thus particularly suitable to conduct behavioral analytic research that study phenomena that are common to all people.

### Development and Features

Back- and front-end development of the casino and AMT integration was outsourced to a professional web development firm. The application relies on C#, ASP.net, Jquery and Bootstrap CSS frameworks, and an SQL database, and features a responsive design suitable for both smartphones, tablets, and computers. Randomness (both stimuli presentation, outcomes, and arm allocation) is implemented through a trial-by-trial random number generator, ensuring random draws with replacement, as in real-life gambling. The validation analyses described below include examining the randomness generation mechanism, since this is crucial to mimicking real-life gambling (4).

**Figure 1:**
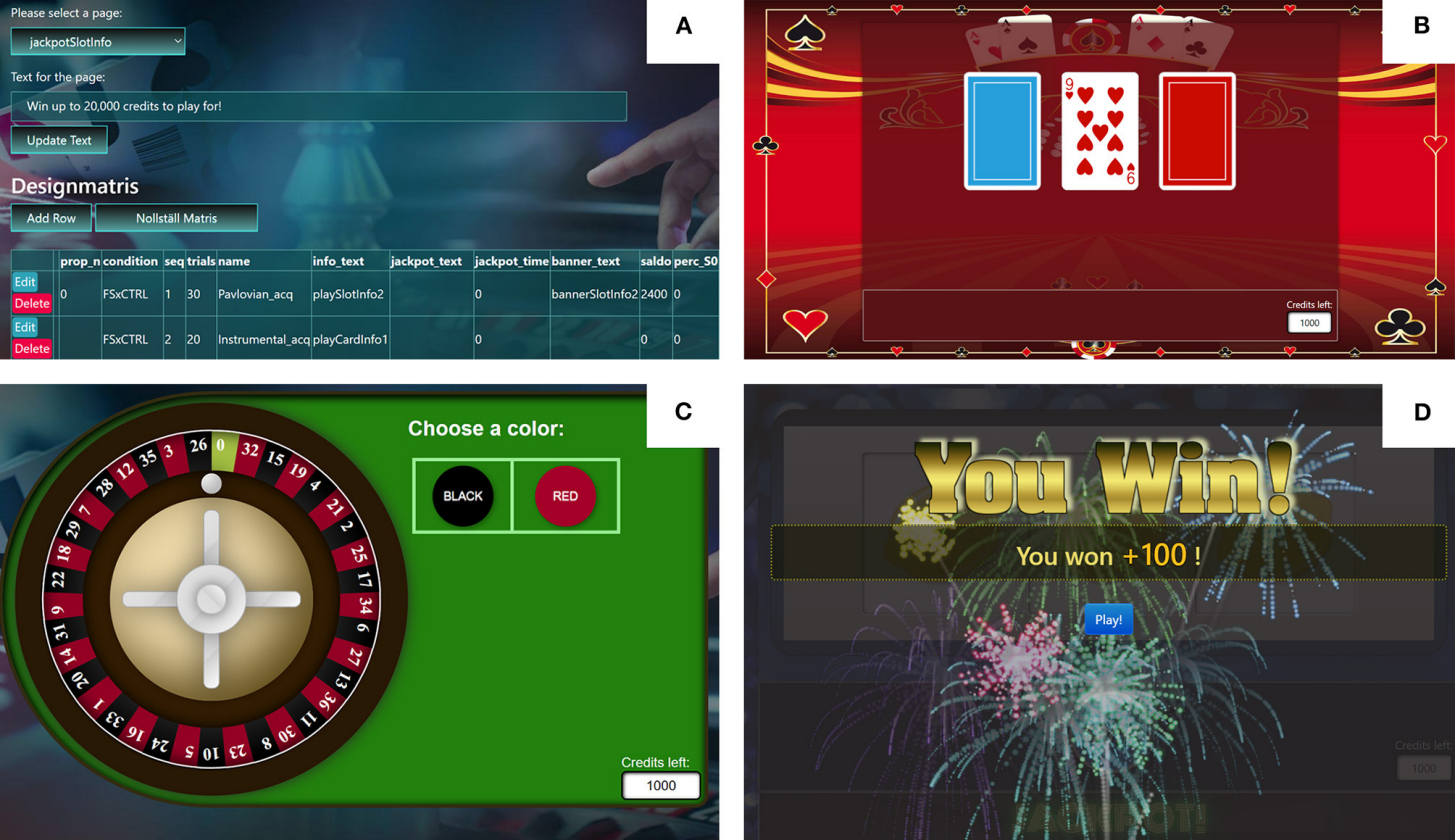
Screenshots of the Frescati Online Research Casino (FORC). **(A)** Administrator view. **(B)** Card-draw game with one visual theme. **(C)** Roulette game with standard visual theme. **(D)** Slotmachine game with another visual theme.

Data from multiple experimental arms can be collected at the same time, with random allocation to arms according to a percentage specified in a design matrix. FORC features three types of games, which can be included in any sequence and with varying number of trials: a roulette wheel with a choice of betting on red or black color (potential instrumental learning task, Figure 1C), a three-reel slot machine with no choice (potential Pavlovian learning task, Figure 1D), and a simple card-choice game with a choice of two decks placed side-by-side either vertically or horizontally (potential instrumental learning task, Figure 1B). While the two former paradigms perfectly mimic real-life gambling, a deliberate design decision was made to not model existing casino card games in order to avoid evoking already learned play strategies that could interfere with the designed contingencies. All games feature realistic sound effects, both on interaction (button pressing) and win outcomes (Figure 1D). Continuous background music was not included due to technical reasons. Balance is by standard displayed in the lower right corner, as in real-life online casinos, but can be hidden by specifying this in the design matrix. Four distinct visual themes—different color schemes, all with graphical casino connotations (one with four variants with only minor differences in element composition)—are available for both the card game and slot machine, which can be randomly allocated per trial. A basic theme option is also available. For each arm, number of trials per sequence, starting balance, visual theme, bet size(s) and win amount(s) and win probabilities, per choice option (if any), can be conveniently set in the design matrix using an online administrator view. See Figure 1A. Short, customizable messages can be displayed in-between games (sequences) to e.g., mimic the sort of messaging used in RGTs (e.g., “Remember that there is no guarantee that you will win back lost credits”) (21).

### AMT and Casino Procedure

Experiments are published on lists of available tasks on AMT; the platform offers the possibility to offer the task only to users with curtained registered characteristics (e.g., country of residence). Task listing includes a short description and reimbursement offered. Interested participants are referred to an AMT landing page featuring a full, customizable description of the experiment, along with participant and informed consent information (see below). Participants consent by clicking on a link that refers to FORC, housed on a separate server. The FORC landing page includes some final instructions, including an emphasis on playing the games as if it were a real working casino. Participants then answer questions on sex, age, last-year gambling frequency (in five steps, from not at all to once a per day or more often, coded 0–4) and types (12 different ones including ones prevalent in non-Western countries, plus a none-option), and the Problem Gambling Severity Index, PGSI (29), a validated screener for gambling problems. Participants then proceed to the games, as dictated by the design matrix. At the end of the games, participants view a customizable message and are shown a custom key, and are then prompted to return to the AMT platform and the key there, which is then used on the AMT side to validate the work performed and approve reimbursement.

### Data Structure

Experimental data are saved and structured trial-by-trial, in long format, and includes anonymous study ID (independent of AMT worker ID), timestamps (temporal resolution was set at seconds at time of collecting validation data, later changed to milliseconds), allocated study arm, game type, trial number, balance in, presented theme, chosen behavior (response), the outcome, and balance out. Survey data can be linked to experimental data through the anonymous study ID generated upon submitting survey data and proceeding to the games. Data can be exported at any time from the administrator view.

### Validation Data

During a roughly 3-h period, *n* = 102 final participants (see below) were recruited from AMT with an offered reimbursement of 2 USD for a session lasting no longer than 30 min. This reimbursement is relatively high compared to the estimated AMT average (30), and would thus likely have made it an attractive opportunity and likely to have promoted high-quality data. The published task description advertised it as a scientific experiment about online gambling. After completing the survey, the experimental setup had participants complete 40 trials of the card-draw game, then 40 trials of the slot machine game, and finally 16 non-reinforced trials of the card-draw game (not used in analyses). While the recruitment aim was *n* = 100 participants, the AMT integration procedure by necessity makes it possible for participants to complete the experimental part without completing the AMT part and being registered as having done this, explaining why the final sample size exceeded the intended. One participant was excluded for not completing all trials. A total of k = 9,696 trials from *n* = 101 participants were thus available for analysis. See Table 1 for participant characteristics.

**Table 1:** Participant characteristics (full sample).

**Variable**	**Mean (SD) or n (of *n* = 101)**
Age	34.89 (10.32)
Male	*n* = 66 (65%)
Any last-year gambling^*^	*n* = 91
*Lottery*	*n* = 63
*Sports betting*	*n* = 22
*Race betting*	*n* = 6
*Cards*	*n* = 0
*Casino slots*	*n* = 41
*Festival*	*n* = 3
*Dice*	*n* = 13
*Online lottery*	*n* = 19
*Online betting*	*n* = 15
*Online cards*	*n* = 23
*Online slots*	*n* = 12
*Other*	*n* = 2
Last year gambling frequency	
*Not at all*	*n* = 10
*A few times*	*n* = 43
*Once a month*	*n* = 23
*Once per week*	*n* = 22
*Once per day or more*	*n* = 3
PGSI score	4.26 (5.46)
PGSI score > 0	*n* = 63

* *Participants could select multiple gambling forms. PGSI, Problem Gambling Severity Index*.

### Analyses

All analyses were conducted in the R (3.6.3) statistical environment. FORC was validated as an experimental platform by considering three aspects: apparent data quality, randomness mechanics and resulting change in average credit balance over time, and psychometric properties of the survey data. Convergent validity of gambling behaviors observed on FORC was not examined since the experimental setup used was not designed specifically to evoke spontaneous gambling behaviors; however, demonstrating validity of the three aspects independently would suggest that an experimental setup designed to do so can be expected to show also convergent validity.

Quality of data was assessed by calculating percentage of participants who in the card-draw part showed no or limited response variation (outside a 10–90% response variation range), indicative of poor data quality due to indiscriminate, repetitive responding; or no such pattern, indicative of satisfactory data quality.

Second, three game mechanics aspects of FORC were empirically evaluated. First, the observed random appearances of gambling outcomes (wins) during the slot machine phase (with different win percentages dependent on random stimulus shown) were compared to those programmed in the design matrix (50% for theme S1A and 20% for theme S2), both on a trial-by-trial basis and overall. Second, to ensure that random draws (outcomes) were made with replacement (i.e. independent of previous ones), we calculated percentage of win outcomes during the instrumental acquisition phase (same 45% win probability in all trials) as a function of outcome of the preceding trial. Third, change in credit balance over time during the slot machine phase (same 20 credit possible win outcome in all trials) was compared to the expected credit balance change based on programmed probability. Since win probability differed between 20 and 50% depending on what stimulus was randomly presented for each trial, a perfect distribution of stimuli across trials and participants would give a 35% win probability. Since each bet cost 10 credits (at time of validation data collection not refunded in case of win; changed after collecting data for the current study, altering only the return to player rate but no game mechanics), and the win outcome was 20 credits regardless of theme, the return to player rate was 0.7 credits, meaning that with perfect distribution of themes between trials, a player's balance should decrease with on average 3 credits per trial.

Third, we performed psychometric analyses on the questionnaire data to estimate quality and validity of the different included measures. Cronbach's alpha (internal consistency) was calculated for the PGSI and factor structure estimated using parallel analysis (31). Associations between PGSI score, gambling frequency and gambling types were also examined using regression models.

### Ethics

The Regional Ethical Review Board in Stockholm has approved the use of FORC for a set of behavioral analytic research studies on gambling behaviors (2018/1968-32 and 2020-01863). Participant information is provided on the AMT platform, after which users can consent by actively choosing to be directed to FORC. In the participant information, it is recommended that potential participants with a history of or current problematic online gambling habits refrain from participation; As of current, it is however not technically possible to exclude participants with high scores on the included PGSI measure completed prior to beginning the experiment. After completing all trials, the end-message is configured to include a statement about the study aims and structure, that any gambling strategies learned in the experiment will not translate into real-life gambling, that the house always wins in real-life gambling, and that participants worried about their gambling habits should seek help locally. For ethical reasons, participant reimbursement is not made contingent on behavior during the experiment (due to e.g., allocation to different win probabilities).

## Results

### Data Quality and Feasibility

During the 40 trials of the card-draw game, no participant showed zero response variation and only *n* = 6 had a response variation outside the 10–90% range, indicative of poor data quality. The remaining *n* = 95 showed greater response variation, with a sample average variation score of 52.4% (SD = 17.7%), i.e., equal response frequencies. Mean completion time was 10.05 min (SD = 3.68), with minimum of 6.35 and maximum of 29.28 min. Examining the duration distributions revealed that only a small minority of participants had durations in excess of 15 min (*n* = 8) and even fewer (*n* = 3) in excess of 20 min. Importantly, a longer duration need not in itself present an issue since the experiment was divided into phases, and participants could have loaded the game and delayed the start. In lieu of any obvious thresholds for determining quality at this level of detail, duration was not considered a quality indicator and hence not used for further exclusion.

### Game Mechanic Validation

**Figure 2:**
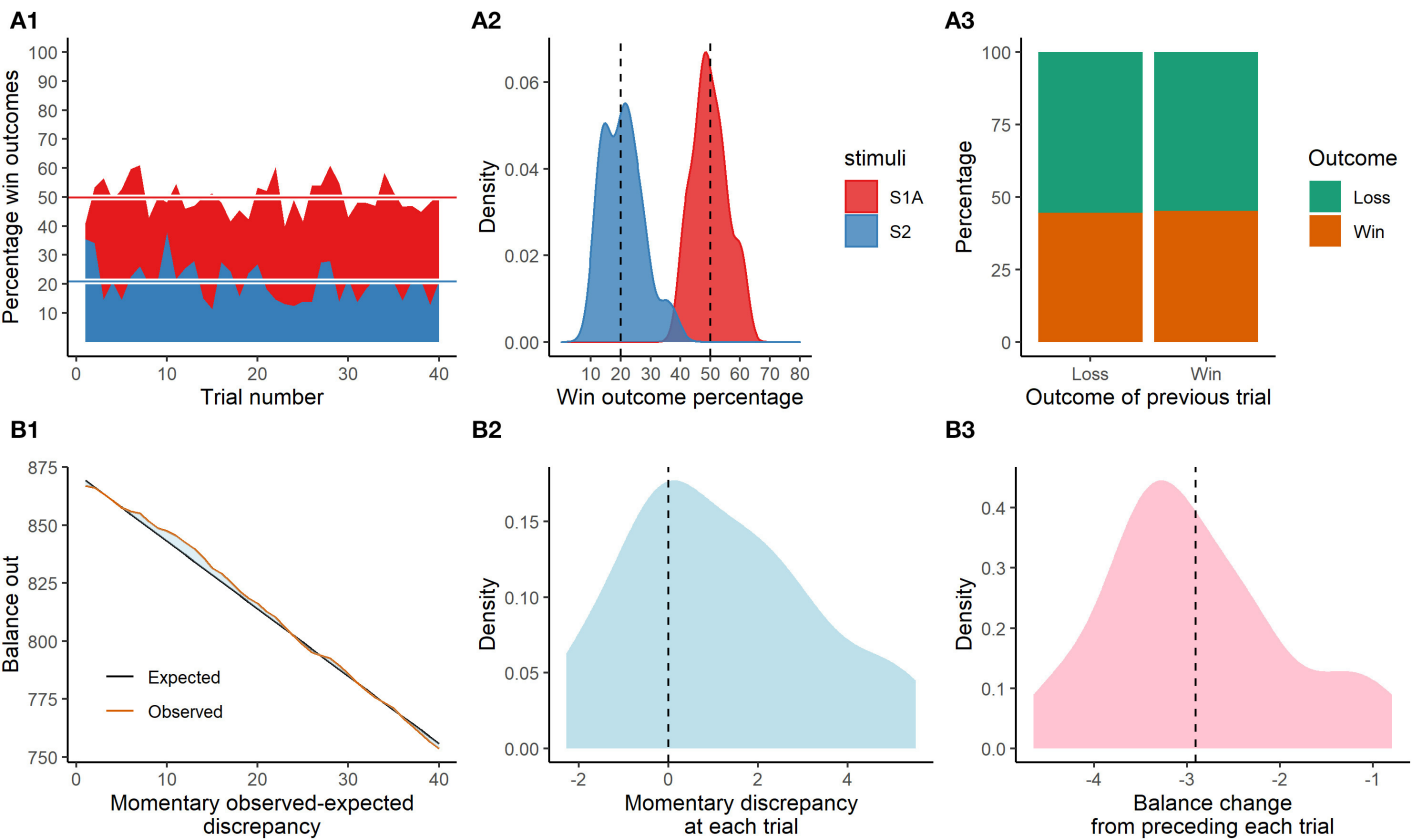
Game mechanics validation results. **(A1)** Percentage win outcomes and distribution thereof **(A2)** across trials depending on randomly displayed theme (stimulus). **(A3)** Win outcomes as function of preceeding trial outcome. **(B1)** Observed balance out across trials compared to expected based on programmed return to player rate. **(B2)** Distribution of observed-expected discrepancies (vertical reference line of zero). **(B3)** Change in balance out as compared to the preceeding trial (vertical reference line corresponds to programmed return to player rate).

Observed win outcome percentages across slot-machine trials were normally distributed at a sample-level around 49.9 and 20.8%, respectively, against set win percentages of 50 and 20%. Observed percentage wins across card-draw trials was 44.5% when preceding trial had a loss outcome, and 45.2% when preceding trial had a winning outcome, revealing that the random mechanism (random sampling with replacement) worked as intended (set win percentage 45%). See Figures 2A1–A3.

Themes were randomly sampled during the slot-machine trials (set probabilities 50–50%), resulting in a 51.5% occurrence of theme S1. This, in combination with the set difference in win percentages between themes (50 vs. 20%), resulted in a total observed win percentage of 35.45% (with perfect 50–50% distribution of themes, the total win percentage would have been 35%, i.e., halfway between 50 and 20%), and in turn an expected credit loss at each turn of −2.911 (which would have been −3 with perfect 50–50% distribution of themes) against a bet of 10 and the equivalent of a return to player rate of 0.71. Observed balance decrease closely followed the expected decrease and was in general normally distributed around it. However, due to a random fluctuation of increased winnings around trial 5–15, and balance being an accumulated measure, the average total momentary expected-observed discrepancy was positively skewed to a mean of M = 1.12 (95% CI: 0.48–1.83). Average balance change from the preceding trial was however a perfect−2.909 (95% CI: −3.22 to −2.60) and normally distributed, revealing that the game mechanics worked as intended when considering that presentations and outcomes were random by design see Figures 2B1–B3.

**Figure 3:**
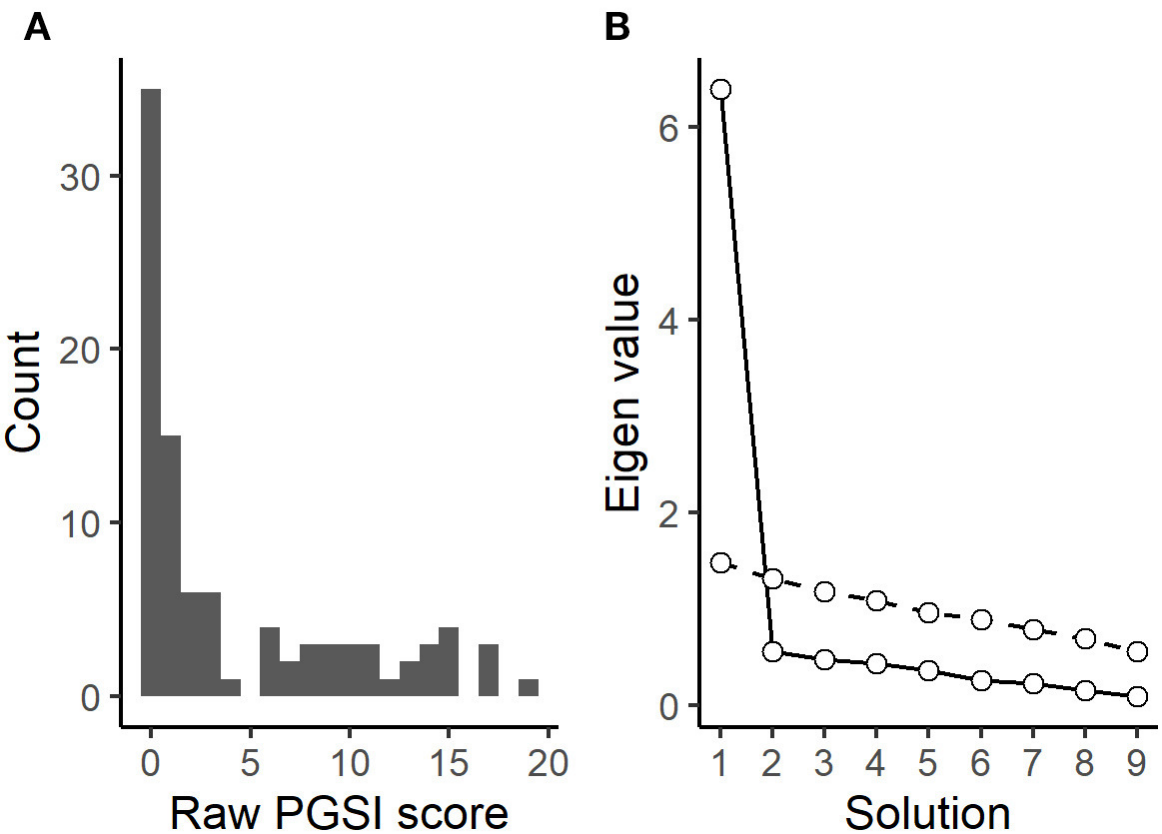
Questionnaire findings. **(A)** Distribution of PGSI scores. **(B)** Parallel analysis screen plot of PGSI items: solid line shows observed values, dashed resampled (comparison).

### Quality and Validity of Survey Data

Quality and validity of survey data was examined among the *n* = 95 who provided quality data in the card-draw game. As expected from a general population sample with an established overrepresentation of problem gamblers (27), PGSI scores were Poisson distributed with excess zeros yet with a long tail. See Figure 3A. Participants who reported no past-year gambling had significantly lower PGSI scores (B = −4.53, SE = 1.88, *p* = 0.0183), and both number of gambling types (B = 0.82, SE = 0.37, *p* = 0.0278) and gambling frequency (B = 2.60, SE = 0.49, *p* < 0.001) were associated with PGSI scores in the expected direction. In a Poisson regression model, gambling frequency significantly predicted number of gambling types (B = 0.34, SE = 0.067, *p* < 0.001).

Cronbach's alpha for the PGSI was calculated to α = 0.95 (95% CI: 0.93–0.96). Even when omitting participants with a PGSI score of zero to avoid artificial inflations of internal consistency due to floor effects (32), α was 0.92 (95% CI: 0.89–0.95). Parallel analysis of PGSI items showed a convincing one-component solution; see Figure 3B.

## Discussion

The Frescati Online Research Casino (FORC) was designed to offer a valuable middle-ground between internal and external validity, providing full and flexible experimental control of a realistic, simulated online casino, in order to study the learning and extinction mechanisms of gambling behavior and evaluate responsible gambling tools and policies in a convenient way. This first validation study showed that data collection through integration with the Amazon Mechanical Turk crowdsourcing platform was feasible, provided a high percentage of high-quality behavioral and survey data, and that the game mechanics worked as intended. This suggests that FORC is ready to be used for experimental studies on gambling behavior and effects of RGTs.

Online gambling is now the most prevalent type amongst problem gamblers (1, 20) (at least in countries where this gambling form is widespread), and can be simulated for research purposes more easily than a traditional casino games since contextual confounders do not apply: participants engage with FORC in the same environment (on their computer or smartphone) that they would with real online gambling. Online gambling as a modality provides better opportunities for behavioral tracking and collecting other data, as well as providing micro-interventions like automated feedback that can all be packaged as part of RGTs (32), making it easier to simulate for research purposes with retained face validity. While there are empirical studies on RGTs (21), most of these have either prioritize internal validity over external validity (e.g., small samples and a laboratory setting), or vice versa (e.g., lack of randomization, allowing no causal conclusions). Deploying experiments via FORC provides a valuable, translational middle-ground that could help to establish an evidence base for RGTs on par with the scientific standards of psychological and medical interventions.

Of note, by both design and current functionality, FORC is limited in some respects as to what types of gambling that can be simulated (see Limitations below). Prominently, we opted to design a new card game—with familiar symbols and general mechanics—to allow the study of instrumental learning, rather than use existing ones, in order to avoid confounding effects of prior learning (i.e., playing styles). The other two FORC games however are very similar to their real-life equivalents, albeit somewhat simpler in gambling options. Of importance to learning experiments, a deliberate design was made to require user input for every trial of the slot-machine, since we considered this to be a key feature of real-life gambling. Although requiring user action to initiate a learning trial deviates somewhat from traditional Pavlovian paradigms, users were presented with only a single option (to continue, i.e., no option to either quit, change bet etc.). According to the so called functional-cognitive framework wherein learning is seen as an ontogenetic adaptation (33), learning in absence of choice can only be Pavlovian and not instrumental.

For ethical reasons, participants with a history of gambling problems are explicitly discouraged from participating. However, it is currently not technically feasible to automatically exclude users with high PGSI scores from participating, for example, or to use this information for arm allocation (although a conditional statement with reference to the PGSI variable would have been easy to add to gate progression from the questionnaire section to games, it would not have hindered participants from simply reloading the page and reporting differently). Not unexpectedly, a large percentage of participants did report at least some gambling problems—even higher than in previous studies using AMT (27), although the international recruitment base make these numbers hard to compare. This observation makes deployment of FORC an ethical issue, rather than theoretically imposing a limitation on generalizability of findings (since little or no selection bias is apparent). As with any research on this topic and/or using similar methods, planned experiments should be vetted by an independent review board. Of importance, FORC includes several features that address this issue directly, including post-experiment debriefing, a reminder that the house always wins, that gambling strategies applied in FORC will not work elsewhere, and encouragements to seek help. Further, considering the ubiquity of online advertisements for gambling opportunities, it could also be argued that presenting AMT users with possibility of participating in a gambling experiments does not in any practical sense increase their exposure to gambling opportunities.

A stated aim of FORC was to offer a wide variety of possible outcome measures, the choice of which must be considered for each particular experiment. Delay in specific responses may be of interest in some experiments (34), yet setting up distinct behavioral choices in the card-draw game, e.g., a high vs. low risk option, may have better convergent validity as a proxy measure of problem gambling and has seen use in past research (35, 36). Whether such measure shows convergent validity will however ultimately depend on the exact experimental setup and must thus be examined in each study carried out using FORC. Of note, another commonly used proxy measures of problematic gambling, gambling persistence (13), is not possible to examine with FORC since AMT participants have no incentive to continue playing beyond the required trials and reimbursement is fixed for both technical and ethical reasons.

The detailed logging procedure featured in FORC also allows for a variety of quality assurance measures. Although AMT experiments do tend to produce high-quality data (26), this does not apply to 100% of participants. In the current study, we examined both within-questionnaire convergent validity and psychometric properties, as well as response variation—the latter on the grounds that fully repetitive gambling would be in violation of experiment instructions and the easiest way to play through the experiment and gain reimbursement as quickly as possible. Response variation is likely to be a sensitive proxy measure of quality, yet possibly at the price of some specificity, and the exact threshold should thus be carefully considered. Since collecting validation data, a new quality assurance feature has been added to FORC in the form of a pop-up question on contingency knowledge acquisition, used in previous research (37). These questions, along with response variation patterns and timing of responses, should be sufficient to make an accurate assessment of data quality in any experimental setup.

Since collecting validation data for the current study, some additional changes have been made to FORC. Win outcomes now always return the bet—this decision was informed by parallel beta testing by other researchers and students (unfortunately, not systematically collected or analyzed), who expressed an expectation from real-life gambling experiences that this was expected. Return of bets upon winning is now explicitly explained in the pre-game instructions, and we can thus see no reason why it would change the game mechanics beyond calculation of the return to player rate, which with one exception (see Limitations below) can easily be adapted. Another change is that bet size, which could previously only be observed through change in the credit balance, is now displayed visually immediately upon pressing a button or selecting a deck, then fading rapidly. Temporal resolution of logged behaviors has been updated to milliseconds to enable computational modeling experiment (34). Additional features added include the possibility to display different messages to different experiment arms at the beginning of each game as per the design matrix, as well as the possibility to add a banner-type advertisement to the background. Both these features were included to be able to study the effects of RGTs like pop-up messaging (21) as well as rule-governed behavior (4).

### Limitations

Both this validation study, and the FORC platform itself, have some limitations that need to be acknowledged. First, the experimental setup was designed to allow a detailed evaluation of the game mechanics, rather than to evoke spontaneous gambling behaviors perfectly reflective of real-life gambling. For example, the return to player rate of the slot machine game was 0.7, which is lower than in typical real-life gambling; although the degree to which participants could discriminate this is unknown (38). For this reason, we refrained from examining associations between observed gambling behavior and collected measures of gambling habits and gambling problems. Instead, we emphasize that each study in which FORC is used should examine convergent validity in relation to what can be reasonably expected given the particular experimental setup used. If, for example, a study aims to immediately promote Pavlovian or instrumental learning in order to avoid possible confounding, the resulting gambling behaviors may be shaped more by the newly learned contingencies than regular gambling strategies, decreasing power to detect convergent associations with survey-reported gambling. Second, the current study did not collect any additional data to examine data quality (e.g., participant ratings or free-text evaluations), opting instead to examine data quality using the same metrics that would be available to subsequent experiments run using the same platform. Importantly, data quality assessment should be carried out in every study that uses FORC, adapted to the specific experimental setup and preferably using pre-registered thresholds. Third, this validation study was not designed to evaluate the optimal description used for recruiting AMT workers to complete the experiment.

While the FORC platform was designed to offer great flexibility in terms of experimental setup, some limitations nonetheless apply. First, although the aesthetic of FORC was designed to mimic that of modern online casinos, graphical quality is not fully comparable, at least to those prevalent in Western countries. To some extent, this was a deliberate design decision: too complex graphical presentations may have distracted participants and presented technical issues for users running the experiment on smartphones and cellular internet connections. Also for technical reasons, including background sound was not possible, although FORC does feature realistic casino sound effects. The impact of lack of background music on external validity remains unknown; although background music during e.g., slot-machine playing may drive immersion and put the gambler in a so called “Dark Flow” (39), gambler may be equally likely to turn down repetitive background music of this kind if they find it disturbing or distracting. A second FORC feature limitation is that only one win probability and amount can be set for each trial sequence, unlike in real-life gambling where there are often several win outcomes available, with probabilities decreasing with increasing amounts. However, jackpot-type setups can still be simulated by setting up several consecutive trial sequences of the same game, with randomized allocation to different number of trials and specific jackpot outcomes if need be. Third, custom gambling options are not available and cannot be simulated at present, meaning that research questions on this particular topic cannot at present be investigated using FORC. Fourth, our subsequent choice to modify the game mechanic to always return the bet on a winning outcome, entails that FORC cannot at current be used to study the losses-disguised-as-wins phenomenon (40). Returning this parameter setting would however require only a minor change to the underlying source code. Finally, it should be acknowledged that as with real-life gambling outcomes, appropriate statistical methods may be necessary to properly analyze some outcomes, e.g., if a particular experimental setup generates an of excess zeroes (41).

## Conclusions

The Frescati Online Research Casino offers a convenient way of performing large-scale experiments on gambling behavior and responsible gambling tools, with an experience resembling real-life online casino gambling. In this first validation study, we show that behavioral and survey data quality appears adequate, and that the game mechanics work as intended.

## Data Availability Statement

The raw data supporting the conclusions of this article will be made available by the authors, without undue reservation. The authors will consider requests for FORC source code.

## Ethics Statement

The studies involving human participants were reviewed and approved by Swedish Ethical Review Authority. The patients/participants provided their written informed consent to participate in this study.

## Author Contributions

PL, JR, and PC designed FORC. EI made significant contributions to design and beta-testing. PL oversaw development, analyzed data, and drafted manuscript. JR, EI, and PC substantial contribution to the interpretation of data and revision of the manuscript for important intellectual content. All authors contributed to the article and approved the submitted version.

## Conflict of Interest

PL is employed by the county-operated addiction healthcare provider in Stockholm and reports funding from the Independent Research Council of *Svenska spel*, a state-operated gambling provider in Sweden. In addition to governmental research grants on gambling, PC reports research funding by several gambling providers in Sweden, Finland and Norway. PL and PC report ongoing academia-industry research collaborations on scientific evaluations of RGTs and impact of the COVID-19 situation on gambling in Sweden. EI reports past research funding from PaF, a gambling provider operated by the Åland Island government. The remaining author declares that the research was conducted in the absence of any commercial or financial relationships that could be construed as a potential conflict of interest.
